# Readiness of Ghanaian health facilities to deploy a health insurance claims management software (CLAIM-it)

**DOI:** 10.1371/journal.pone.0275493

**Published:** 2022-10-05

**Authors:** Gordon Abekah-Nkrumah, Maxwell Antwi, Alex Yao Attachey, Wendy Janssens, Tobias F. Rinke de Wit

**Affiliations:** 1 Department of Public Administration and Health Services Management, University of Ghana Business School, University of Ghana, Legon, Accra, Ghana; 2 Management Department, Pharm Access Foundation, East Legon, Accra, Ghana; 3 Research and Innovartions Department, Pharm Access Foundation, East Legon, Accra, Ghana; 4 Amsterdam Institute for Global Health and Development, School of Business and Economics, Vrije Universiteit, Amsterdam, The Netherlands; 5 PharmAccess Foundation and Department of Global Health, Amsterdam University Medical Centre, University of Amsterdam, Amsterdam, The Netherlands; Yale University, UNITED STATES

## Abstract

**Introduction:**

Inadequate, inefficient and slow processing of claims are major contributors to the cost of health insurance schemes, and therefore undermining their sustainability. This study uses the Technology, Organisation and Environment (TOE) framework to examine the preparedness of health facilities of the Christian Health Association of Ghana (CHAG) to implement a digital mobile health insurance claims processing software (CLAIM-it), which aims to increase efficiency.

**Methods:**

The study used a cross-sectional mixed method design to collect data (technology and human capital capacity and baseline operational performance of claims management) from a sample of 20 CHAG health facilities across Ghana. While quantitative data was analysed using simple descriptive statistics statistics (frequencies, mean, minimum and maximum values), qualitative interviews were recorded, transcribed and abstracted into two major themes that were reported to re-enforce the quantitative findings.

**Results:**

The quantitative results revealed challenges including inadequate computers and accessories, adequate numbers and skills for claims processing, poor intranets and internet access, absence of a robust post-implementation support system and inadequate standard operating procedures (SOPs) for seamless automation of claims processing. In addition to the above, the qualitative results emphasised the need to make CLAIM-it more flexible and capable of being integrated into third-party softwares. Notwithstanding the challenges, decision-makers in CHAG health facilities see the CLAIM-it software as having better functionality and superior capabilities compared to existing claims processing systems in Ghana.

**Conclusion:**

Notwithstanding the challenges, the CLAIM-it software is more likely to be adopted by decision-makers, given the positive perception in terms of superior functionality. It is important that key actors in claims management at the National Health Insurance collaborate with relevant stakeholders to adopt the CLAIM-it software for claims processing and management in Ghana.

## Introduction

Achieving universal health coverage (UHC) constitutes a key objective of health policy in both developed and developing countries [1]. Efforts to improve UHC are paramount especially in developing countries where disease burden and mortality are high. In response to this, Ghana introduced a National Health Insurance Scheme (NHIS) in 2003 to reduce financial barriers to health service utilization [2]. NHIS reached 38% coverage in 2013, 39% in 2016, and 40% in 2020 [3]. The scheme covers a wide range of diseases (about 95%) in Ghana and exempts several groups (children under 18, those above 70 years of age, pregnant women, contributors to the Social Security and National Insurance Trust (SSNIT) and those who are extremely poor) from paying premium. The sources of funds for the NHIS are 2.5% Value Added Tax on vatable transactions, 2.5% of contributions of the SSNIT Fund, premium paid by members of the scheme, registration fees as well as interest on funds invested by the National Health Insurance Authority (NHIA) [4].

Since the establishment of the NHIS, the scheme has adopted different methods for purchasing services from providers in an attempt to improve on efficiency and reduce costs. The NHIS started with a fee-for service (FFS) arrangement in 2005. In 2008, the diagnostic-related group system of payment, known as the Ghana Diagnostic-Related Grouping–G-DRG system was introduced [3, 4]. Besides the G-DRGs, NHIS also piloted a capitated payment scheme in the Ashanti region in 2012. Although the G-DRGs and the capitation system (currently abandoned) were supposed to increase efficiencies under the FFS, costs rather escalated [4]. Although this increase is partly attributed to growth in membership of the scheme and therefore utilization, supply and demand side moral hazards have also been identified as major causes of the increased costs [5–7].

A key contributor to high health insurance costs is the processing of claims. This ranges from overbilling of medicines, inappropriate application of tariffs, duplication of claims, lack of evidence on diagnosis to back claims, absence of a link between treatment and diagnosis, treatment outside the defined benefits package, irrational prescription of medicines, inflation of the quantities of medicine supplied to subscribers, provision of services above accreditation level and overbilling of medicines [4, 8]. This has contributed to financial losses to the NHIS and thus is threatening the scheme’s sustainability. Also, these issues contribute to delays in claims processing and payment, leading to decreased trust and participation by both patients and providers, as well as provider adaptive mechanisms that have adverse implications for availability and quality of care [7].

To address these challenges, the NHIA in collaboration with PharmAccess Group developed a software (CLAIM-it) to manage claims processing. CLAIM-it is a four-module application, comprising a claims entry module, a receiving (aggregation) system, a claims adjudication module and a Regional and District Health Director’s reporting module. The **claims entry module** implements and enforces all the necessary claims generation rules and protocols of the NHIA. Hence claims submitted for adjudication are validated by the software and this ensures due diligence prior to claims submission. The claims entry module runs fully offline and thus allows users to work independent of the internet. It can be installed and operated on a single user computer or implemented on a network, with as many user nodes as needed. It can also be integrated into any existing Hospital Management Systems (HMS).

Claims submission takes into account the lack of internet in some parts of Ghana and the distance travelled by some facilities to submit their claims to Central Processing Centres (CPCs). Claims generated via CLAIM-it can be submitted either directly to NHIA over the internet or with data downloaded and saved on a flash drive for submission at a local district NHIA office or CPC. Submitted claims are aggregated in the **receiving system,** and using a newly developed provider coding system, are grouped according to the region, district, type of facility and facility ownership, before distribution to the various CPCs for adjudication and reporting. Reports generated by the receiving system before and after adjudication are available to both the NHIA and respective Regional and District Directors through an **online portal**. The reports provide an overview of the types of claims submitted by providers, the total volume of claims submitted, cost associated with claims submitted and submission date of the claims. Regional and District Directors of Health can use the information provided through the reporting system to plan. It is anticipated that providers in the future will be able to track progress of their claims from submission through adjudication to payment. Thus, CLAIM-it is expected to tackle inefficiencies in claims generation, claims collation, claims storage and claims submission and vetting. With the NHIA claims vetting protocols built into the CLAIM-it software, it is expected that providers will experience reduced delays in claims processing and the NHIA will also be able to reduce time to vet claims. The CLAIM-it software was piloted in a few CHAG health facilities and is currently being scaled up to the full CHAG network of morev than 300 health facilities. This study examined the readiness of CHAG health facilities to implement CLAIM-it. Particular attention was paid to human resources capacity (numbers and skill sets), technological preparedness (hardware and relevant infrastructure readiness status of selected health facilities), claims generation and submission, benefits of the CLAIM-it application and potential implementation challenges. Results are put in the context of the operational performance of existing claims management systems, with emphasis on claims output (the value of monthly claims and deductions as well as the average number of days it takes to submit claims).

### Relevant literature

It is widely acknowledged that the use of Information Technology (IT) has noteworthy effects on the productivity of businesses [9]. Organizational readiness is a major factor in implementing a new technology such as the CLAIM-it application. The Technology Organisation Environment (TOE) framework [10] was used as a conceptual model to examine the readiness of health facilities to implement the CLAIM-it application, based on it wide applicability across geography and industries including; manufacturing [11], healthcare [12], retail and financial services [13]. The TOE framework [14] suggest that three contexts (technological, organizational and environmental) influence processes adopted by a firm to implement a technological innovation [14]. The technological context describes current practices and equipment internal to the firm as well as the set of available technologies external to the firm [15, 16]. Organizational context refers to the scope, size, resources and managerial structure of the organization that constrain or facilitate use of technology [17, 18]. The environmental context is the arena in which a firm conducts its business,—its industry, competitors, and dealings with the government [19, 20].

The technological context has been represented by several variables such as relative advantage, complexity, IT infrastructure, IT expertise [16]. Relative advantage can be defined as the extent to which an innovation or a technology is perceived as offering an advantage over previous ways of performing the same task [21]. Relative advantage can be measured in terms of performance and convenience. Literature suggests that organizations are more likely to implement a new technology if the technology can bring about better organizational performance and higher economic gains [22]. Complexity is defined as the degree to which an innovation or technology is regarded as relatively difficult to understand and use [23]. If technology is seen to be complex by employees, it negatively influences adoption or implementation of that technology [18]. IT infrastructure is defined as collection of physical technology resources which provide the basis for IT-related purposes [24]. Readiness to implement technology depends on availability of IT infrastructure [25]. Additionally, IT expertise, which refers to employees’ knowledge of using technologies [26] positively influences an organisation’s likelihood of implementing new technology-related applications [27].

Also, firm size constitutes an important factor that influences organizational performance and also flexibility to implement technology [28]. Large firms, unlike small firms have more resources and hence are likely to adopt and implement a new technology faster [29]. However, in some instances, small firms were swift and flexible in implementing technology, especially when financial and technological resources are controlled for [30]. A firm’s scope (the geographical extent of an organization’s operations) is also associated with a higher likelihood of technology adoption and implementation [20, 30, 31]. Finally, the need to out-perform competitors [32], support from an organization’s top management [33] and regulators [26] as well as financial and human resources [34] have all been shown to positively influence the adoption of a new technology.

Although the TOE framework is applicable to technology adoption in diverse contexts (electronic data interchange [35]; open systems [36]; web site [10]; enterprise resource planning [37] and business to business e-commerce [38]), specific contexts are associated with unique sets of factors that influence adoption [39]. Factors that have been identified as determinants of adoption are technology competence, firm size, global scope, enterprise integration, competition intensity and regulatory environment [40], IT infrastructure and management, government regulation and promotion [41].

On the basis of the above literature, we argue that human resource capacity, available technological infrastructure, existing processes for generating and submitting claims, knowledge of potential benefits and possible implementation challenges of the CLAIM-it application as well as output from the existing claims management system constitute key factors that will enable or constrain adoption and implementation of the CLAIM-it application by health facilities.

## Methods

### Study design

A cross-sectional multi method design was used to address the objectives of the study through the use of survey that was subsequently augmented by inerviews with staff.

### Sampling

The study focused on CHAG health facilities in Ghana that had not yet implemented the CLAIM-it software. Health facilities fulfilling this criteria were 115 out of a total of 333. These comprised hospitals (37), Health Centres/Primary Health Centres/Community Health Planning and Services- CHPS (HCPHC-CHPS) (11), and Clinics (67). A four-criterion process was used to select a representative sample from the 115 health facilities. First, the sample size was set at 20 health facilities, deemed sufficient to reach information saturation as per previous studies [42]. Second, to achieve geographic representativeness, Ghana’s three geographic zones were used to allocate the 20 health facilities proportional to the number of health facilities in each zone. This meant 10, 4 and 6 health facilities for the Middle, Northern and Coastal zones respectively. Third, types of health facilities were selected proportional to the number of each type of health facility found in each geographic zone. Thus, 5 hospitals, 4 clinics and 1 HCPHC-CHPS were selected in the Middle zone; 2 hospitals and 2 clinics were selected in the Northern zone, and 3 hospitals, 1 clinic and 1 HCPHC-CHPS were selected in the Coastal zone. Physical accessibility of providers was considered as a requirement for selection into the sample. Finally, selection of specific providers was done to capture the widest possible range of variation in responses.

### Data collection

Following from the literature review, readiness of health facilities to deploy the CLAIM-it software is argued to be contigent on available human resource capacity, technological infrastructure, sound existing processes for generating and submitting claims, knowledge of potential benefits (including coparative strengths of existing claim management software and the CLAIM-it software) and possible implementation challenges of the CLAIM-it software. Thus, data collected through a survey using closed and open ended questions as well as semi-structured interviews focused on the above. Whiles the survey focused on on facility preparedness for the deployment of CLAIM-it (technological capcity and human capital capcity); claims generation and submission and baseline operational performance of the claims-management unit of each health facility (value of claims and deductions), the interviews focused on claims generation and submission and knowledge of CLAIM-it and potential challenges. Each health facility answered one questionnaire which contained two sections; survey and interview guide. Before data collection, a draft of the questionnaire was pre-tested using two CHAG health facilities in Accra that did not participate in the data collection process. The pre-tested questionnaire was amended to reflect comments from the pre-testing. The questionnaire (both survey and interviews sections) were administered by two trained enumerators (a female and male PhD candidates at the time of data collection, who did not have any relationship with the facilities or officers interviewed) to three staff members of each health facility whose roles are related to the claims processing functions of the health facility (the accountant, an officer of the hospital in-charge of claims processing and the administrator). Although the three officers were restricted to different sections of the survey (sections mainly relevant to their work), they were interviewed together to enable triangulation of earlier answers and/or to ask follow-up questions. The interviews were audio-recorded to enable discussions arising from follow-up questions to be captured. Beside triangulation, the interview re-enforced findings from the survey.

### Analysis of data

To examine implementation readiness, the human resources and technological capability of the Claims Unit of the health facilities studied were examined. In addition, the claims generation and submission process, knowledge on the CLAIM-it software as well as potential implementation challenges were also examined. Operational performance of the claims management system, with emphasis on claims output (the value of monthly claims and deductions as well as the average number of days it takes to submit claims) was studied, both as a basis to establish whether the CLAIM-it application has an advantage over existing systems and also as a basline for follow-up evaluations.

Simple descriptive statistics (frequencies, mean, minimum and maximum values) were used to analyse the survey (human resource and technology preparedness, claims generation and submission and claims output). To better understand the quantitative findings, a qualitative analytical approach was used to re-enforce findings from the survey. Specifically, recorded data were transcribed into an MS-Word document. The transcripts were compared to the audio tape to ensure that data on the tape was captured accurately. Information from the transcripts were abstracted into seven themes but captured under two broad themes in the results (claims generation and submission and knowledge of CLAIM-it and potential implementation challenges) and constituted the basis for presenting the results.

### Ethical consideration

Ethical approval was received from the Ethical Review Committee of CHAG with serial number 190001. Management of each participating health facility gave a written administrative approval for the study to be conducted in their health facility. In addition to the administrative approval, participating officers gave verbal consent and were informed that they could withdraw from the study at any time without any consequence whatsoever.

## Results

### Profile of health facilities

The profiles of health facilities assessed for the study are shown in Table 1.

**Table 1:** Profile of health facilities studied.

Variable	NHF	Mean	Min	Max
Facility size in terms of bed capacity	20	68	10	250
Total facility workforce	20	154	21	483
Average patient turnover in facility	20	3,534	300	11,382
Size of annual revenue (GH₵)	17	2,840,000	24,000	11,000,000
Value of facility assets (GH₵)	10	4,160,000	153,000	15,500,000
Number of years in operation	20	30	5	88

NB: The value of the dollar to the Ghana Cedi (GHC) is USD 1 to GHC 5.7; patient turnover is annual and NHF is number of health facilities

The average health facility bed size was 68 (maximum 250; minimum 10), less for rural areas than urban centers (results not shown). The workforce averaged 154 workers (minimum 21; maximum 483), with health facilities in urban areas having a relatively higher average (192) compared to the average in rural areas (84). Patient turnover averaged 3,534 patients a year (minimum 300; maximum 11,382). The turnover in rural areas was 2,074 patients, which was twice as low as the average patient turnover in urban areas. Out of the 20 facilities examined, 17 gave information related to their annual revenue. The average annual revenue is GH₵2,840,000 (about USD 50,000) with minimum and maximum revenue figures of GH₵24,000 and GH₵11,000,000 respectively (Table 1). Average revenue in rural facilities was much lower than their counterparts in urban areas. Also, 10 out of 20 of the health facilities reported the current value of their assets, which averaged GH₵ 4,160,000, with rural areas having a much lower value (GHC 1,290,000) compared to urban areas (GHC 6,080,000). Health facilities on the average had operated for 30 years, with a maximum of 88 years, minimum 5 years. Consistent with the current trend, urban hospitals have on the average been in operation for a much longer period (32 years) compared to rural health facilities (26 years). Finally, 11 of the health facilities examined do not use a health management information system, with 5 of them located in an urban area and 6 in rural areas (results not shown).

### Human resource capacity for claims management

In this sub-section we present results on human resource (HR) capacity (see Table 2) as relevant for claims processing, to assess whether HR would provide sufficient manpower for implementing the CLAIM-it software.

**Table 2:** Human resource capacity for claims management.

Variable	Obs	Mean	Min	Max
Total number of staff working in the claims unit	20	4	1	8
Total staff who work in the claims unit as permanent employees	20	3	0	6
Total staff who work in the claims unit as casual employees	20	1	0	3
Number of claims staff responsible for claims vetting	20	2	0	6
Number of claims staff involved in JUST data entry	20	2	0	6
Number of claims staff trained in MS-Office	20	2	0	7

The data collected indicate that an average of 4 staff work in the Claims Management Unit, with the least number of staff being 1 and a maximum of 8. Additionally, 3 out of the 4 staff in the Claim’s Unit are permanent staff with 1 being casual. In terms of Claim’s Unit staff responsible for vetting, an average of 2 are responsible for this, with maximum of 6 and a minimum of 0 (i.e. no person solely assigned to claims), with about 2 workers on the average involved in data entry (maximum 6) and trained in MS-Office applications (maximum 7). In terms of usage, 79%, 53% and 21% of the staff using MS-Office applications indicated that they use Word and Excel, PowerPoint and Outlook and MS Project respectively.

### Technological preparedness for CLAIM-it

Table 3 presents information on technological resources of health facilities that may be relevant for the adoption of an electronic claims processing system such as CLAIM-it.

**Table 3:** Technological resources for claims management.

Variable	Obs	Mean	Std.Dev.	Min	Max
**Panel A: Number of Computers**					
Number of functional computers /laptops/tablets in the facility	19	23	27.64	1	85
Number of functional computers in this facility solely dedicated to claims data entering and processing	20	3.05	1.79	0	6
Number of functional surge protectors in this facility solely dedicated to claims data entering and processing	20	1.05	1.63	0	6
**Panel B: Availability of LAN and Accessories**					
**Variable**			**Response**	**Freq.**	**Percent**
Do you have a local area network in place (LAN)			Yes	10	50
			No	10	50
Do you have in place an arrangement to support your hardware and LAN			Yes	11	55
			No	9	45
Do you have in place backup power systems in time of power failure			Yes	12	60
			No	8	40

Health facilities owned an average of 23 computers (minimum of 1 and maximum of 85). On the average, 3 functional computers were dedicated to claims processing, with some health facilities recording a maximum of 6 whiles others did not have any dedicated computers for data processing at all. Also, there was an average of 1 functional electrical surge protector in the health facilities studied, although some had as many as 6, with others having none. In addition, 50% (10) of health facilities in the study had local area networks (LAN), 55% (11) had arrangements to support their hardware and LAN, with 60% (12) having backup power systems in place.

### Claims generation and submission

Table 4 presents information on claims processing procedures to assess potential CLAIM-it implementation readiness.

**Table 4:** Frequency distribution of claims generation and submission.

Variable	Response	Freq.	Percent
Does this facility have a written guideline or manual SOP for capturing/entering data and processing claims	Yes	5	25
	No	15	75
If YES request to obtain or see a copy	Copy obtained	1	20
	Copy requested and seen	3	60
	Copy requested but not available	1	20
Is this facility a credentialed NHIS service provider	Yes	20	100
	No	0	0.00
If YES request to obtain or see a copy of credentialed certificate	Copy requested and seen	12	60
	Copy requested but not available	8	40
If YES, please state where your claims are submitted to for processing.	District Office	3	15
	CPC	17	85
In what form do you usually submit your claims	Paper only	2	10
	Electronic only	10	50
	Both paper and electronic	8	40
Does this facility keep claims data electronically	Yes	19	100
	No	0	0

Thus, 5 out of 20 health facilities had written guidelines or manuals (Standard Operating Procedures, SOP) for capturing or entering data and processing claims. From the 5 with guidelines or SOPs, 1 gave a copy of the SOPs out, 3 showed a copy of the SOPs, with 1 unable to show a copy of their SOPs. Furthermore, all the health facilities studied were credentialed NHIS service providers. However, only 12 of them were able to present documentation to this effect. Additionally, 3 of the health facilities submit their claims to the district office for processing, while the rest (17) submit their claims to the Central Processing Center.

With respect to the submission process, 10 used electronic only submission process, followed by both electronic and paper (8) and paper only (2). In terms of the storage media used by the health facilities, all the health facilities kept their claims electronically, mainly using storage media such as a dedicated server, computer hard disk, external hard drive, CD or pen drive. Overall, 5 of the health facilities surveyed used a dedicated server, 1 health facility used dedicated server and external hard drive, 8 used computer hard disk, 1 used computer hard disk, CD and pen drives, 1 used computer hard disk, CD, pen drives and external hard drives, 2 used CD or pen drives and 1 used external hard drives. In addition to storing data electronically, 13 health facilities used off-the-shelve software and MS-Excel to generate and process claims, with the remaining using other software.

The results of the qualitative data suggest that claims management is made up of three stages: generation of the base data, processing and validation of the data and finally submission of the data to the NHIA. Two systems (manual and electronic) are used for the generation of base claims data. However, irrespective of the system used, the qualitative results indicate that the validation and processing of claims is done electronically, either using MS-excel, an off-the-shelve or a bespoke software, with most of the vetting and processing protocols automated. The submission is mostly done electronically as already demonstrated in the quantitative results, with only a few health facilities submitting manually as per the quote below.

*“For claims we normnally have data from our transactions which has already been captured into our HIS*. *That data is entered into another software which helps us to validate the claims for inclusion in the claims to be submitted for the month*. *We then submit the claims report ot NHIA for reimbursement”*. *………………*. *R4*

More importantly, inherent challenges were identified with the existing system(s) for generating and submitting claims. For example, those who use a manual system experienced challenges such as (1) the length of time it takes to process claims, (2) the fact that the process is error ridden (due to diagnosis mismatch, lack of access to pricing information) and (3) equally expensive due to the cost of inputs (personnel and other inputs) used. For those who use an electronic system to generate the base data and then use a bespoke software to validate and process claims, the main complaint was inadequate computers and accessories like power back-ups and lack of appropriate technical support from vendors as per the quote below.

*“Validating claims for submission can be a tedious job*, *especially if you have to enter the data into your health insurance software manually”………You will have too many mistakes*, *leading sometimes to the rejection of claims and it takes all your time and delays the submission of the calims to NHIA”…………………*…*Respondent 5*

*“Using an automated claims management system has been helpful interms of reducing errors and the time it takes to compile and validate claims………*..*Our main challenge is computers*, *power buckups and and maintenance support”…………………*..*Respondent 2*

The qualitative results further suggest that internet access is a major challenge, especially given that it is needed to access the NHIA system to generate a unique code for patients. For those who had just started using the CLAIM-it software (testing), a major source of frustration was the inflexibility of the software and the lack of thorough training to help users to navigate the software.

*“The CLIAM-it software is very good*, *but the problem is that it is not flexible*. *This is a hospital and sometimes you have an emergency where you will not be able to do things as expected*. *So it should be possible for the software to allow you to do want you have to do and later make the corrections if needed*. *Else we will loose our clients……they are mostly unhappy when yu say the software is nnot making it possible”*. *………………………*…*Respondent 13*

### Knowledge of CLAIM-it and potential implementation challenges

The qualitative interviews revealed that almost all of the respondents knew of CLAIM-it’s existence, with some respondents participating in specific training programmes. Respondents were unanimous that CLAIM-it is potentially superior to current systems being used. The main areas of expected benefits mentioned were: reduction in errors due to the inbuilt NHIA validation protocols, speed in claims processing, cheaper alternative for processing claims due to the fact that there is no need to print the claims, thus reducing cost of paper. Health facilities also indicated that the possibility of online claims submission means one may not need to physically go to the Central Processing Center, a potential cost saver. Some respondents also suggested that CLAIM-it can reduce the level of stress of staff in the claims unit. Additionally, the respondents indicated that with CLAIM-it, one is able to have access to detailed information on the claims submitted, which can be used for management decision-making. The above findings is captured in the quotes below.

*“I will prefer using the CLAIM-it software because it has the NHIA protocols as part of the software*. *In addition*, *you can submit the claims online to the CPC and this saves time and can save paper from printing”…………*… *Respondent 16*

*“The fact that the CLAIM-it software can be used with our current software*, *for me is a game changer*. *This means that we can just import from our software and CALIM-it can help is to process and validate claims in a short time”……………*. *Respondent 3*

Notwithstanding the benefits articulated above, majority of the respondents indicated that there could be challenges with the roll-out and implementation of CLAIM-it, with the following constituting major areas of concern:

- The need for adequate computers and accessories, reliable local area networks and internet.
- The need to for adequate personnel who have the skill to use computers for claims processing. In this regard, adequate training was emphasised.
- Given that CLAIM-it adheres to the NHIA vetting protocols, the software will in most cases not allow users to proceed unless the transactions being processed adhere strictly to the respective NHIA vetting protocols. Hence potential users suggested the need to ensure that CLAIM-it is flexible enough to allow for the processing of certain transactions even though they may not meet the vetting protocols at the time of processing. To them what is most important is that they can later correct whatever anomalies before finally submitting such claims.
- The need to ensure that there will be adequate technical support to the users of CLAIM-it so that their technical challenges can easily be addressed.
- The need to find a way to integrate CLAIM-it into existing software that generates the base data for claims processing. The challenge raised was that manually entering information that is available in electronic formats from third-party software into CLAIM-it is tedious and time consuming.

### Claims output

In this sub-section, we present results on the performance of the current claims management system in terms of the output it delivers and this includes the value of monthly claims and deductions as well as the average number of days it takes to submit claims.

### Value of monthly claims and deductions

Table 5 presents descriptive statistics of the number and value of total monthly claims from July 2018 to June 2019.

**Table 5:** Average value of total monthly claims (July 2018 to June 2019).

Variable	Obs	Mean	Min	Max
July 2018	20	100,000	3,018	431,000
August 2018	20	103,000	1,856	428,000
September 2018	20	94,064	2,006	351,000
October 2018	20	104,000	2,801	383,000
November 2018	20	106,000	2,685	389,000
December 2018	20	93,212	1,835	317,000
January 2019	20	98,480	1,767	371,000
February 2019	20	94,106	1,686	330,000
March 2019	20	104,000	1,842	363,000
April 2019	20	110,000	1,871	388,000
May 2019	20	123,000	2,188	414,000
June 2019	18	123,000	2,503	404,000

**Fig 1:**
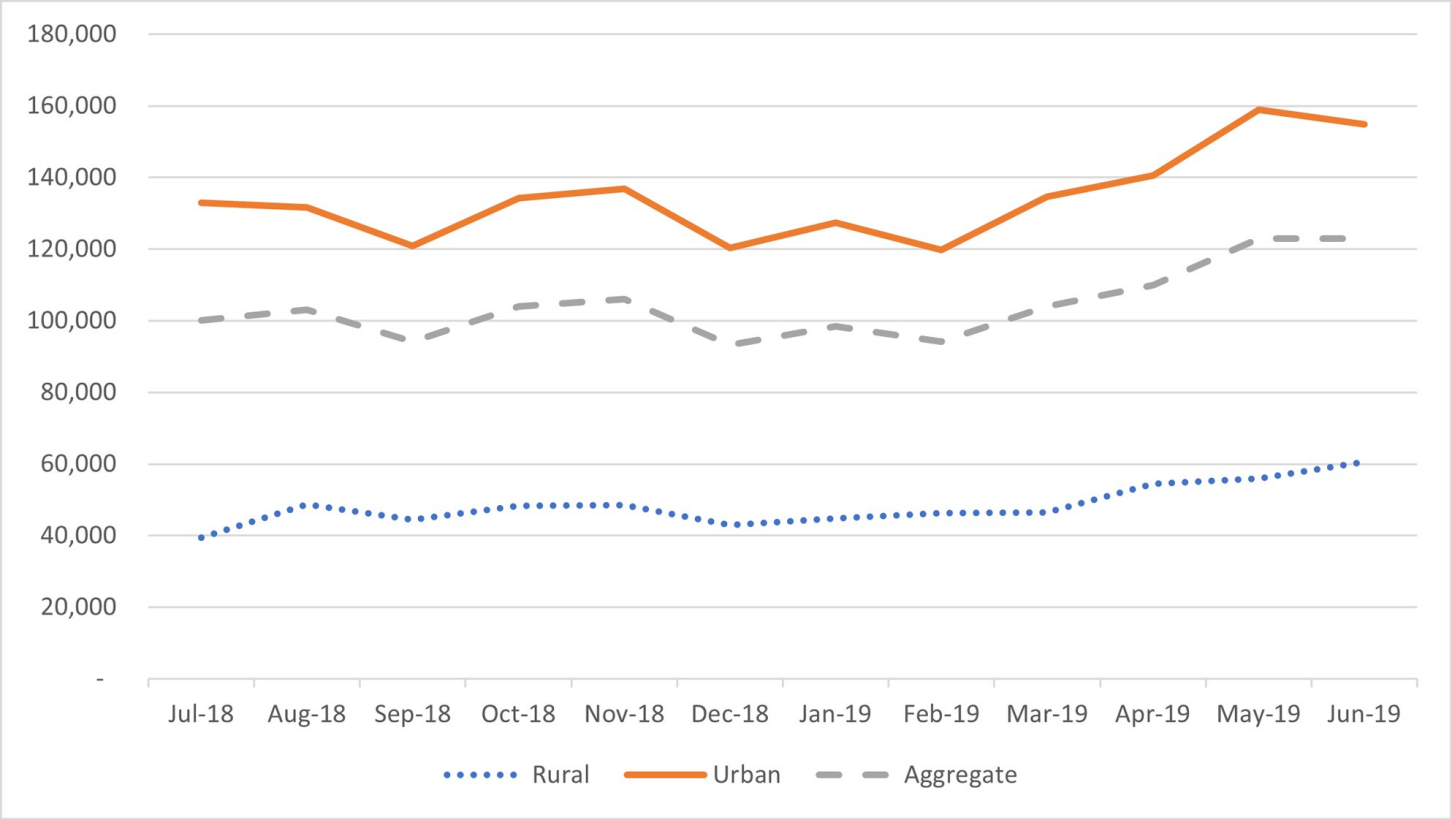
Comparison of average value of total monthly claims in Ghana Cedis. (Please, see figure file for Fig 1).

May and June 2019 recorded the highest average monthly claims of GH₵123,000. Additionally, Fig 1 indicates that the average value of monthly claims has been increasing since February 2019, with the rate of increase being much sharper in urban areas compared to rural areas. Whereas the highest urban average monthly claim was GHC 158,935 in May 2019, that for rural health facilities was GHC 60,563 in June 2019.

**Fig 2:**
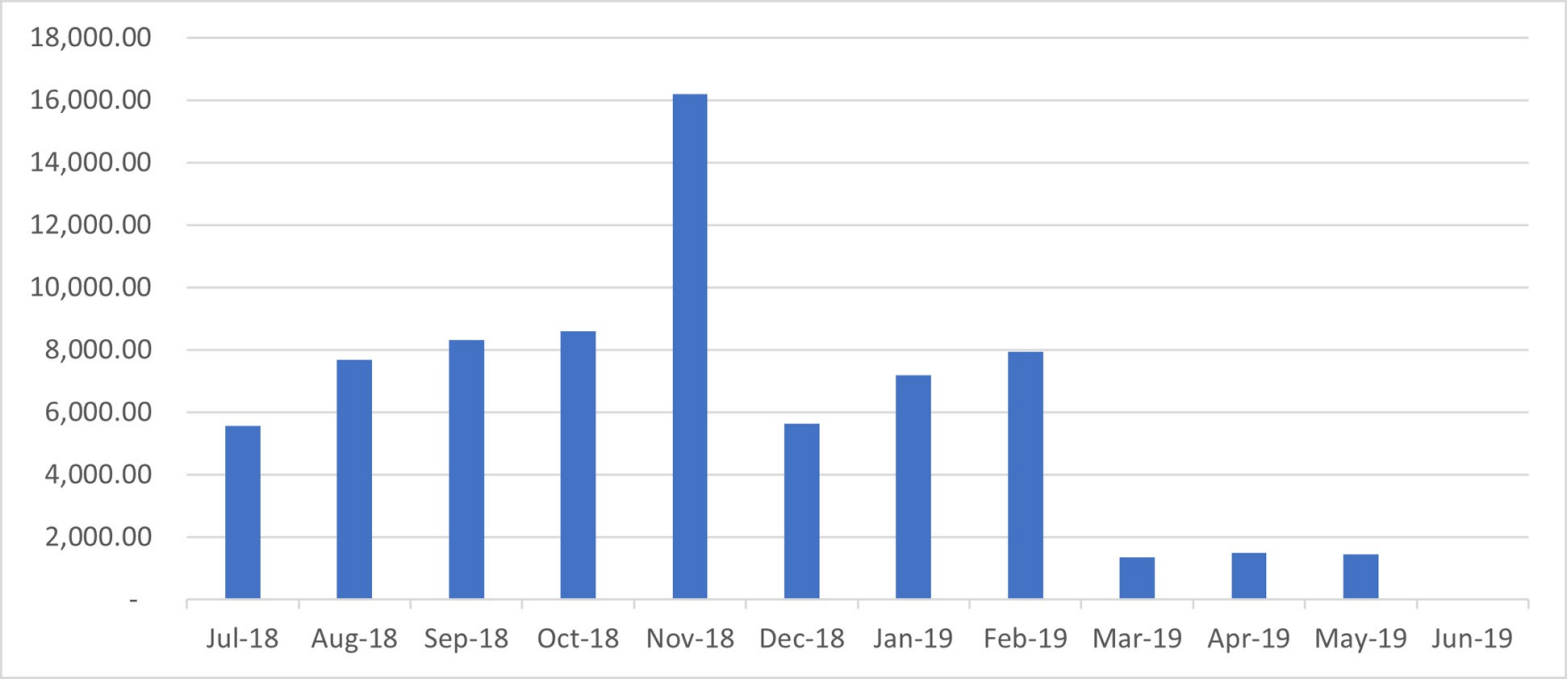
Average monthly deduction from submitted claims in Ghana Cedis. (Please, see figure file for Fig 2).

Fig 2 shows data on average monthly deductions by the NHIA on incorrect claims submitted from July 2018 to June 2019. The number of health facilities that provided information from July 2018 to December 2018 were 10 while less than 10 facilities provided information from January 2019 to May 2019. In terms of average deductions, November 2018 recorded the highest average of GHC16,187. The next highest average deduction of GHC8,608 was in October 2018. In terms of absolute numbers, the highest and lowest deduction of GHC 146,000 and GHC 6 respectively, occurred in November 2018. Beside claims revenue and deductions, the data collected indicate that 18 of the health facilities surveyed have at one point in time had errors in claims submitted and 17 of them had their claims ever rejected by the NHIA. The results (not shown) indicate that it took an average of 4.6 days to submit claims to the NHIA office between July 2018 to December 2018, fluctuating between 1–20 days. However, this changed to 2.7 days, with a minimum and maximum of 1 and 10 days respectively for January 2019 to June 2019.

## Discussion

This study using multi method approach and a sample of CHAG health facilities, sought to examine the readiness of the health facilities to implement the CLAIM-it software. Core findings of the study cover five key domains (HR capacity, claims generation and processing, technological preparedness, claims output and potential benefits and implementation challenges of CLAIM-it).

The quantitative results suggest that on the average there were sufficient staff at the Claims Unit for the purpose of processing claims even though for some health facilities this was not the case. It is important to reiterate that the size of health facilities interviewed (the highest of which are district hospitals, which normally will not be up to 100 beds in size) are such that an average of 4 staff for the Claims Unit should be enough for the work they do. The majority of staff in the Claims Unit are computer literate, suggesting that it will be relatively easy to use existing software or adapt to a new software [27]. These observations imply that the CLAIM-it software is unlikely to have difficulties in the area of human resources when adopted by CHAG health facilities.

On the contrary, technological preparedness, a key input into technology adoption readiness [15, 16, 25] seems to be low. There will be the need for increased investments in computers and accessories, reliable local area networks (LAN) and access to the internet which per the qualitative interviews seem to be crucial both for accessing the NHIA system and also sending data to the NHIA. These investments will be crucial for the success of CLAIM-it implementation, given that efficient functioning of the CLAIM-it software will depend on the availability of adequate number of appropriate computers and accessories, LAN and a properly functioning internet.

The TOE framework suggests that managerial structures can facilitate the adoption of new technology [17, 18]. An example of such structures that can aid the implementation of the CLAIM-it software are SOPs for claims processing. The results indicate that only a few of the health facilities interviewed (5) have such SOPs in place. There is the need to build capacity in this area. Given that 18 of the health facilities interviewed use some form of electronic systems (10 electronic only and 8 a combination of paper and electronic), we argue that any attempt to implement the CLAIM-it software successfully will depend on the extent to which implementers will leverage on the capabilities of users of the existing electronic claims processing systems. Additionally, the fact that existing claims management processes are error ridden and that it takes an average of 4.6 days (up to 10 days) to submit claims, indicates that healthcare facilities are currently putting up with problematic claims management systems. It is important to emphasise that while such challenges may constitute a catalyst for managers who want to be competitive in the market place to abandon existing problematic systems (Oliveira et al., 2014) and adopt CLAIM-it as an alternative, it is equally essential to point out that that such weak structures can limit the ability of managers to implement CLAIM-it successfully when adopted.

Given that performance and convenience (measures of relative advantage within the TOE framework) [21, 22] and flexibility [18, 23] have been advocated to positively influence technology adoption and implementation, it is important to address the issue of inflexibility that has been identified with the CLAIM-it software. Thus, introducing some flexibility in enforcing the NHIS protocols as well as the integration of the CLAIM-it software into third-party applications used to collect the base claims data will be essential. Although users appreciate that enforcing the NHIS protocols will reduce errors in claims processing, and therefore reduce the likelihood of claims being rejected by the NHIA, they are equally concerned that enforcing the NHIS protocols in the context of their business processes may inconvenience a lot of their patients. Thus, finding a balance between accuracy and flexibility will be key in adopting and implementing the CLAIM-it software. Additionally, while perceived superior capabilities and positive view of the CLAIM-it software compared to existing substitutes will increase the likelihood of adoption [21, 22], its integration into third party applications will reduce the amount of required data entry and make the use of CLAIM-it attractive compared to existing alternatives.

For claims output, the results indicate that monthly claims revenue are on the increase while the level of monthly deduction as well as the time it takes to submit claims to the NHIA office is has reduced over the last year. Although it is not directly apparent from the current data what may be causing the trend of positive claims outcomes in recent months, it is possible that our observed increased use of electronic systems by some of the health facilities may be partly responsible. Thus, it is possible that the full implementation of the CLAIM-it, viewed as a more superior software compared to existing available softwares will lead to further improvement in claims processing and consequently claims output.

We emphasize that although attempts were made to attain a representative sample of health facilities (20 out of 115 CHAG facilities that have not yet implemented CLAIM-it), the possibility of sampling bias cannot be ruled out entirely. Thus, results may not necessarily reflect full generality of the situation of all CHAG health facilities. However, the qualitative information gathered from the 20 facilities confirms our quantitative information, strengthening the validity of our results. Additionally, the focus of the current study was on preparedness and so did not cover user experiences of the very few who have tested the CALIM-it software. A large portion of the technology adoption literature has focused understanding factors that facilitate or constraint adoption with issues of readiness not explicitly emphaised. Thus, the findings of the current study adds to the adoption literature by additionally emphaising the importance of readiness (required HR capacity, required structures and processes to aid adoption, technological preparedness in terms of appropriate infrastructure and assesories, perception of benefitds and challengesassociated with adoption) to technology adoption.

## Conclusion

This study examines readiness of health facilities to implement the CLAIM-it software. The study used the TOE framework, that emphasises technological, organisational and environmental factors as key to the adoption and implementation of new technologies. The results suggest that the adoption and implementation of the CLAIM-it software can be a challenge to several of the health facilities studied, due mainly to low technological preparedness (inadequate computers and accessories, poor intranets and internet access). Additionally, the absence of a robust post-implementation support system, challenges with the claims processing capabilities of existing claims processing systems, inadequate SOPs for a seamless automation of claims processing and the required integrations between existing claims processing software and the CLAIM-it software may pose challenges. The above challenges notwithstanding, health facilities studied tend to have a positive view of the CLAIM-it software, and perceive it to have superior functionality and capability compared to existing systems used to process claims. Such a positive view of the CLAIM-it software can facilitate and encourage of adoption of CLAIM-it.

Thus, to ensure that implementation is successful, it will be important for stakeholders (CHAG secretariate, NHIA, PharmAccess and most importantly the health facilities) to work together. Key in this regard will be providing health facilities the right computers and accessories, especially backup power systems, LAN and appropriate internet access. Although our findings indicate that human capital does not constitute a major challenge for implementing CLAIM-it, it will remain important for health facilities to continue training their existing staff not only for operating the software but also providing relevant technical support to users. Also important will be to continue addressing operational deficiencies identified in the CLAIM-it software (e.g. integration into other third party applications already in use and post-implementation support), making available requisite structures and procedures for automating the claims processing function. Finally, there will be the need to recalibrate the CLAIM-it software and make it more responsive to the needs of health facilities. The ability to balance accuracy with flexibility will be crucial to adoption and therefore implementation. It is anticipated that readiness for the implementation of CLAIM-it will be enhanced substantially if the issues above are addressed.

## Supporting information

S1 File Survey questionnaire and qualitative interview guide. Data collection instrument has information on profile of health facilities, HR capacity for claims management, claims generation and submission, claims output and qualitative interview guide.
(DOCX)

S2 File Data. Anonymized data as per the content of attached quantitative instrument.
(XLSX)

S3 File Data. Anonymized data as per the content of attached interview guide.
(XLSX)
