# Supplementary material for: Readiness of Ghanaian health facilities to deploy a health insurance claims management software (CLAIM-it)
Source: PLoS One. 2022 Oct 5;17(10):e0275493. doi: 10.1371/journal.pone.0275493 (PMC9534449; doi:10.1371/journal.pone.0275493)
Supplement: S1 File — Data collection instrument has information on profile of health facilities, HR capacity for claims management, claims generation and submission, claims output and qualitative interview guide. (DOCX) [file pone.0275493.s001.docx]

**Survey Questionnaire and Qualitative Interview Guide**

*In the facility, administer this questionnaire to the head, administrator and person in charge of generation and submission of claims.*

Name of the Health facility: ………………………………………………………………............

Name of the district: ………………………… Nam e of Region: ………………………

Date of interview: …………………………………………………………………………

Name of interviewer: …………………………………………………………………

Name of person(s) interviewed:

1………………………………………………………Function ………………………….

2………………………………………………………Function…………………………

3………………………………………………………Function………………………….

1.Tel…………………………………………………Email ……………………………….

2.Tel…………………………………………………Email……..…………………………

3. Tel…………………………………………………Email…….………………………….

**Health Facility Profile**

1. What is the size of your hospital in terms of bed capacity?
2. What is the total workforce of the hospital?
3. What is the average patient turnover in your hospital?
4. What is the size of your annual revenue in Ghana cedi terms?
5. What is the current value of the assets of the hospital in Ghana cedi terms per the most recent financial statement?
6. For how many years has this hospital been in operation?
7. Is the Hospital currently using any form of Hospital management software?

Yes No

1. Please specify the type of software being used and its purpose

-------------------------------------------------------------

1. What is the geographic location of the hospital in terms of rural or urban?

**Human Resource Capacity for Claims Management**

1. Name and designation of officer responsible for claims processing in this facility?

Name Designation

------------------------------------------------------- ----------------------------------------

1. Currently, what is the TOTAL number of staff working in the claims unit in this facility?
2. Currently, how many of the TOTAL staff who work in the claim’s unit are permanent employees?
3. Currently how many of the TOTAL staff who work in the claims unit are casual employees?
4. Currently how many of the claims staff are responsible for claims vetting?
5. Currently how many of the claims staff are involved in JUST data entry?
6. Currently how many of the claims staff have been trained in MS Office applications
7. If STAFF have been trained in MS office applications, please choose from the list of applications from the office suite staff have been trained on.
   1. Microsoft Word Yes No
   2. Microsoft Excel Yes No
   3. Microsoft Power Point Yes No
   4. Microsoft Outlook Yes No
   5. Microsoft Project Yes No

**Technological Preparedness for Claims-it**

1. How many functional computers. /laptops/tablets do you have in this facility?
2. How many functional computers in this facility are solely dedicated to claims data entering and processing?
3. How many functional surge protectors in this facility are solely dedicated to claims data entering and processing?
4. Do you have a local area network in place (LAN)? Yes No
5. Do you have in place an arrangement to support your hardware and LAN? Yes No
6. Do you have in place backup power systems in times of power failure? Yes No.

**Claims Generation and Submission**

1. Does this facility have a written guideline or manual (SOP) for capturing/entering data and processing claims? Yes No
2. If YES request to obtain or see a copy.

A) Copy obtained B) Copy requested and seen C) Copy requested but not available D) Other (Specify)

1. Is this facility a credentialed NHIS service provider?

Yes No

1. If YES request to obtain or see a copy of credentialed certificate.

A) Copy obtained B) Copy requested and seen C) Copy requested but not available D) Other (Specify)

1. If YES, please state where your claims are submitted to for processing.

District Office CPC

1. In what form do you usually submit your claims?

A) Paper only B) Electronic only C) Both paper and electronic

1. If this facility also submits claims electronically, which software is used in capturing the data?

Bespoke Off-the-Shelve Excel Others: Specify--------------------------------

1. Does this facility keep claims data electronically? Yes No
2. If this facility keeps claims data electronically, where do you store your claims data?

A) Dedicated server B) Computer Hard disk C) CD/Pendrive D) Other – Specify…………………..

1. What is the reason for the stated electronic claims storage preference?
2. What is the number and value of total monthly claims in this facility from July 2018 to June 2019

| **Month** | **Value of Claims in GHC** |  | **Month** | **Value of Claims in GHC** |
| --- | --- | --- | --- | --- |
| July  2018 |  |  | January  2019 |  |
| August  2018 |  |  | February 2019 |  |
| September 2018 |  |  | March  2019 |  |
| October  2018 |  |  | April  2019 |  |
| November 2018 |  |  | May  2019 |  |
| December 2018 |  |  | June  2019 |  |

1. What is the value of total monthly deductions from claims in this facility from July 2018 to June 2019 due to either errors or inconsistencies?

| **Month** | **Value of Claims Deductions GHC** |  | **Month** | **Value of Claims Deductions GHC** |
| --- | --- | --- | --- | --- |
| July  2018 |  |  | January 2019 |  |
| August  2018 |  |  | February 2019 |  |
| September 2018 |  |  | March  2019 |  |
| October  2018 |  |  | April  2019 |  |
| November 2018 |  |  | May  2019 |  |
| December 2018 |  |  | June  2019 |  |

1. Does this facility have any experience where errors were sometimes detected in submitted claims?

Yes No

1. If yes, please state the main errors that were detected in the submitted claims?

List:

1. Does the facility have any experience of NHIS submitted claims ever being rejected?

Yes No

1. If YES, please state the reasons for the rejection?

---------------------------------------------------------------------------------------------------------------

---------------------------------------------------------------------------------------------------------------

---------------------------------------------------------------------------------------------------------------

**Claims output (“additional benefits”) data**

1. Using the current claim cycle, please indicate below how many days it took to compile and generate claims for each of the following months

| **Month** | **Days for Claims Compilation and Generation** |  | **Month** | **Days for Claims Compilation and Generation** |
| --- | --- | --- | --- | --- |
| July  2018 |  |  | January 2019 |  |
| August  2018 |  |  | February 2019 |  |
| September 2018 |  |  | March  2019 |  |
| October  2018 |  |  | April  2019 |  |
| November 2018 |  |  | May  2019 |  |
| December 2018 |  |  | June  2019 |  |

1. Using the current claims cycle as the reference point, please indicate how long in days it takes you to submit claims generated to the NHIS Claim office

| **Month** | **Days for Claims Submission** |  | **Month** | **Days for Claims Submission** |
| --- | --- | --- | --- | --- |
| July  2018 |  |  | January 2019 |  |
| August  2018 |  |  | February 2019 |  |
| September 2018 |  |  | March  2019 |  |
| October  2018 |  |  | April  2019 |  |
| November 2018 |  |  | May  2019 |  |
| December 2018 |  |  | June  2019 |  |

1. On the average, how many days does it take for you to pay your suppliers of medicines after their invoice is due?
2. On the average, how many days does it take for you to pay your suppliers of consumables after their invoice is due?
3. On a scale of 1 to 6 with **1=strongly disagree, 2=disagree 3=somewhat disagree, 4=somewhat agree, 5=agree** and **6=strongly agree**,

indicate your preferred response to any of the statements below:

| **No** | **Activity** | **1** | **2** | **3** | **4** | **5** | **6** |
| --- | --- | --- | --- | --- | --- | --- | --- |
| 24.1 | Our existing claims management processes makes it convenient to compile and generate claims |  |  |  |  |  |  |
| 24.2 | Our existing claims management processes makes it possible to compile and generate monthly claims in the shortest possible time |  |  |  |  |  |  |
| 24.3 | Our existing claims management processes makes it possible to reduce the number of errors in submitted claims to barest minimum |  |  |  |  |  |  |
| 24.4 | Because of the quality of information produced by our existing claims management processes, the NHIA claims office finds it easy to vet and validate our claims |  |  |  |  |  |  |
| 24.5 | Because of the quality of information produced by our existing claims management processes, the NHIA claims office is unlikely to reject any of our submitted claims |  |  |  |  |  |  |
| 24.6 | Because of the quality of claims data submitted to the NHIA claims office our reimbursement hardly delays |  |  |  |  |  |  |
|  | We are able to use our validated claims as collateral to secure private commercial loans |  |  |  |  |  |  |
|  | We know of the Medical Credit Funds (MCF) and how it can help our hospital in terms of finances |  |  |  |  |  |  |
|  | We have access to the MCF and know what to do to access funds from MCF |  |  |  |  |  |  |
|  | Delays in processing of our claims is a constraint to our ability to access the MCF |  |  |  |  |  |  |
| 24.7 | Our existing claims management processes makes it possible for us to produce routine clinical data (Diagnosis, medicines consumption and patient turnover) |  |  |  |  |  |  |
| 24.8 | Our existing claims management process has made it possible for routine clinical data to be processed into a from that can easily be used for clinical decision-making |  |  |  |  |  |  |
| 24.9 | Because of the existing claims management processes, we are able to use processed clinical information for everyday clinical decision-making |  |  |  |  |  |  |
| 24.10 | Our claims management processes have made it easy for us to generate different types of report (clinical and managerial) useful for managers of the hospital |  |  |  |  |  |  |
| 24.11 | Our claims management processes have made it easy for us to generate different types of report (clinical managerial) useful for district managers of the health system |  |  |  |  |  |  |
| 24.12 | Because of the existing claims management processes, we are able to use information for everyday clinical decision-making |  |  |  |  |  |  |

**Qualitative Interview Guide**

1. Can you describe the process used by your health facility to compile claims?
2. What are the challenges in the current claims compilation and management process?
3. What do you think should be done to resolve what you consider to be challenges in the current claims compilation and management system? Probe further for detailed discussion of the solutions identified and how they work
4. In what ways will the solutions that you have proffered above resolve the challenges you have identified?
5. Have you heard of the CLAIM-it software developed with the help of the PharmAccess Group?
6. If the respondent is not aware of the CLAIM-it software, explain to the respondent what the software is about
7. Find out from the respondent if the CLAIM-it software can be a solution to their challenges and if so how?
8. Find out if there are any constraints that can inhibit the implementation of the CLAIM-it software.
9. Find out from the respondents whether the current system of claims compilation and management makes it possible for them to have access to relevant claims information for decision-making
10. Find out from the respondent what they anticipate to be the benefit of the implementation of the CALIM-it software in their health facility.
11. Ask follow-up questions on the benefits enumerated by the respondents.
